# Diagnostic accuracy and utility of three dengue diagnostic tests for the diagnosis of acute dengue infection in Malaysia

**DOI:** 10.1186/s12879-020-4911-5

**Published:** 2020-03-12

**Authors:** Zhuo Lin Chong, Shamala Devi Sekaran, Hui Jen Soe, Devi Peramalah, Sanjay Rampal, Chiu-Wan Ng

**Affiliations:** 1 Department of Social and Preventive Medicine, Faculty of Medicine, University of Malaya, Kuala Lumpur, Malaysia; 2 Institute for Public Health, National Institutes of Health, Ministry of Health, Setia Alam, Selangor Malaysia; 3 Faculty of Medicine and Biomedical Sciences, MAHSA University, Jenjarom, Selangor Malaysia; 4 Department of Medical Microbiology, Faculty of Medicine, University of Malaya, Kuala Lumpur, Malaysia

**Keywords:** Dengue, Dengue diagnosis, Rapid diagnostic test, Biosensors, Evaluation

## Abstract

**Background:**

Dengue is an emerging infectious disease that infects up to 390 million people yearly. The growing demand of dengue diagnostics especially in low-resource settings gave rise to many rapid diagnostic tests (RDT). This study evaluated the accuracy and utility of ViroTrack Dengue Acute - a new biosensors-based dengue NS1 RDT, SD Bioline Dengue Duo NS1/IgM/IgG combo - a commercially available RDT, and SD Dengue NS1 Ag enzyme-linked immunosorbent assay (ELISA), for the diagnosis of acute dengue infection.

**Methods:**

This prospective cross-sectional study consecutively recruited 494 patients with suspected dengue from a health clinic in Malaysia. Both RDTs were performed onsite. The evaluated ELISA and reference tests were performed in a virology laboratory. The reference tests comprised of a reverse transcription-polymerase chain reaction and three ELISAs for the detection of dengue NS1 antigen, IgM and IgG antibodies, respectively. The diagnostic performance of evaluated tests was computed using STATA version 12.

**Results:**

The sensitivity and specificity of ViroTrack were 62.3% (95%CI 55.6–68.7) and 95.0% (95%CI 91.7–97.3), versus 66.5% (95%CI 60.0–72.6) and 95.4% (95%CI 92.1–97.6) for SD NS1 ELISA, and 52.4% (95%CI 45.7–59.1) and 97.7% (95%CI 95.1–99.2) for NS1 component of SD Bioline, respectively. The combination of the latter with its IgM and IgG components were able to increase test sensitivity to 82.4% (95%CI 76.8–87.1) with corresponding decrease in specificity to 87.4% (95%CI 82.8–91.2). Although a positive test on any of the NS1 assays would increase the probability of dengue to above 90% in a patient, a negative result would only reduce this probability to 23.0–29.3%. In contrast, this probability of false negative diagnosis would be further reduced to 14.7% (95%CI 11.4–18.6) if SD Bioline NS1/IgM/IgG combo was negative.

**Conclusions:**

The performance of ViroTrack Dengue Acute was comparable to SD Dengue NS1 Ag ELISA. Addition of serology components to SD Bioline Dengue Duo significantly improved its sensitivity and reduced its false negative rate such that it missed the fewest dengue patients, making it a better point-of-care diagnostic tool. New RDT like ViroTrack Dengue Acute may be a potential alternative to existing RDT if its combination with serology components is proven better in future studies.

## Background

Dengue is an emerging infectious disease endemic to more than 100 tropical countries [1]. An estimate of up to 390 million dengue infections happened in the year 2010 alone, of which only a quarter were detectable [2]. Dengue threatens a pandemic with the spread of Aedes mosquito, the vector that carries the pathogen dengue virus that comes in four serotypes (DENV1–4), to subtropical regions throughout the world [3].

In Malaysia, dengue incidence stood as high as 396.4 cases/100,000 population with case fatality rate ranging from 0.20–0.28% in recent years. In 2015 alone, dengue virus infected up to 120,000 people and caused 336 deaths [4]. Despite an absence of medical treatment to date, early disease recognition and timely intervention with proper fluid management and supportive care can prevent mortality due to dengue infection [5].

The obstacle to early dengue diagnosis lies in its diverse and unspecific clinical symptoms that resemble other diseases, which leads to delay in health-seeking and misdiagnosis of the disease [1, 6]. Laboratory tests such as virus isolation (VI), reverse transcription-polymerase chain reaction (RT-PCR), haemagglutination inhibition (HI), and enzyme-linked immunosorbent assay (ELISA) for the detection of dengue non-structural antigen-1 (NS1) or dengue-specific immunoglobulin (IgM/IgG) can confirm diagnosis. But they are resource-intensive and not suitable for low-resource settings [7].

The growing demand for point-of-care diagnostics gave rise to many dengue rapid diagnostic tests (RDT) that flooded the market in the past two decades [7, 8]. Recent development in biosensors for the rapid diagnosis of acute dengue infection, if proven accurate, may intensify the competition and make RDT more affordable to more patients who need it [9]. In this evaluation study, we aimed to estimate the diagnostic accuracy and utility of a newly developed biosensors-based dengue NS1 assay, and one each of commercially available NS1 ELISA and NS1/IgM/IgG RDT.

## Methods

### Study design

This evaluation study employed a prospective cross-sectional design. Its reporting followed Standards for the Reporting of Diagnostic Accuracy Studies (STARD) guidelines [10].

### Ethical statement

The study adhered to the principles of the revised 2013 Declaration of Helsinki [11] and obtained ethical approval from the Medical Research and Ethics Committee, Ministry of Health Malaysia (NMRR-17-853-34,393) and University Malaya Medical Center (MRECID.NO: 2017426–5171). Written informed consent was obtained from each participant and minor assent where appropriate.

### Population

The study site was Shah Alam Section 7 Health Clinic, a public clinic with the highest number of dengue patients located in the dengue-endemic district of Petaling, Selangor state, Malaysia. Febrile patients aged 9 months and above with symptoms fulfilling World Health Organisation (WHO) 2009 criteria for suspected dengue that sought treatment at this clinic from 13th November 2017 to 30th March 2018 during normal working hour were recruited consecutively [6]. Patients in need of emergency care or with pre-existing conditions that were prone to complications from blood sampling were excluded. Both capillary and venous blood samples were collected from each patient using EDTA tubes for immediate onsite index tests. Another venous sample in plain tube was also collected together, centrifuged, aliquoted, and stored at − 80 °c for laboratory-based diagnostic tests in a virology laboratory in University of Malaya, Malaysia. No convalescent sample was taken. Socio-demographic background and clinical history were captured using structured questionnaire through face-to-face interview at the same setting. The 500 target sample size calculated using single proportion sample size formula based on a 50% disease prevalence was expected to achieve 5% absolute precision with 95% confidence for both sensitivity and specificity estimates [12].

### Index tests

ViroTrack Dengue Acute (BluSense Diagnostics, Denmark), SD Bioline Dengue Duo and SD Dengue NS1 Ag ELISA (Standard Diagnostics, Korea) were evaluated in this study for comparison. The first two are RDT intended for point-of-care use. They were tested onsite on both capillary and venous blood samples by medically trained research assistants blinded to the clinical pictures of the research participants. Additionally, ViroTrack Dengue Acute was also tested onsite on the same day with serum samples extracted from the plain tubes.

#### ViroTrack Dengue Acute

ViroTrack Dengue Acute (BluSense Diagnostics, Denmark) is a biosensors-based semi-quantitative immuno-magnetic agglutination assay packed in a polymer centrifugal microfluidic cartridge. Its diagnostic mechanism was detailed out previously [13]. Briefly here, for each test, a ViroTrack micofluidic loaded with 30 mcl of blood sample was inserted into a portable opto-magnetic reader – the BluBox. The sample was centrifuged, metered, and mixed with magnetic nanoparticles (MNPs) pre-coated with anti-dengue antibodies located within the cartridge. Dengue NS1 antigen, if present, formed sandwich agglutination with these MNPs and were forced to rotate under an oscillating magnetic field, which modulated the intensity of a laser beam passing through them. A photodetector with a Blu-ray optical pickup unit would then measure the phase difference between the modulated light transmission and the applied field, which corresponded to the level of dengue NS1 antigen. This measurement was presented in a relative unit and interpreted by the BluBox according to a pre-set threshold value, where positive was defined as > = 27, negative if < 23, and equivocal (EQ) if 23–26.9 unit. The whole process after the insertion of the microfluidic was automatic and the result was ready in less than 15 min. The results were recorded by one research assistant and verified by three others independently. For analysis, a patient was considered tested positive for ViroTrack Dengue Acute if either capillary or venous sample was positive, EQ if both were EQ, and negative for all other combinations. No repetition was done for EQ results.

#### SD Bioline Dengue Duo

This is a commercially available rapid immunochromatographic test that comes in a combo of two joint cassettes, one for NS1 and another for IgM/IgG. Only 100 mcl blood sample was needed for the NS1 assay, while serology required 10 mcl followed by assay diluent. Results were interpreted according to manufacturer’s instruction by two independent research assistants 15–20 min after the application of specimen, where appearance of a test line was considered positive in the presence of a control line. Presence of only control line was considered negative [14]. Discrepancies between first and second interpreters were resolved with the help of a third interpreter. For analysis, a patient was considered tested positive to an assay on SD Bioline Dengue Duo if either capillary or venous sample was found positive, and negative if both were negative.

#### SD Dengue NS1 Ag ELISA

SD Dengue NS1 Ag ELISA (Standard Diagnostics, Korea)*,* a commercially available direct sandwich ELISA, was performed together with reference tests and interpreted according to manufacturer’s instruction [15]. Test was considered valid if the negative and positive controls absorbance values were within set ranges. Cut-off value was calculated by adding 0.3 to the mean absorbance for negative controls. A sample was considered positive if its absorbance was equal to or larger than the cut-off value, and negative if lower.

### Reference standard

The reference tests comprised of iTaq Universal SYBR Green One-Step real-time RT-PCR (Bio-Rad Laboratories, Hercules, CA), Panbio Dengue Early ELISA, and SD Dengue IgM and IgG capture ELISA (Standard Diagnostics, Korea). They were performed according to the manufacturers’ instructions as described in detail previously [16–19]. They were chosen in reference to a previous study [20]. These tests were conducted from 6th December 2018 to 13th April 2019, up to around 1 month from sample collection, by trained laboratory personnel blinded to the clinical information and results of the point-of-care index tests. A laboratory-confirmed dengue was defined as 1) RT-PCR positive, or 2) Panbio NS1 ELISA positive; while a presumptive dengue tested negative for both the above, but positive for IgM ELISA [20]. Both laboratory-confirmed and presumptive dengue were included in the analysis as dengue positive; while patients who did not fall into any category above were taken as dengue negative without further laboratory tests. On top of that, a combination of “Recife” method and IgM/IgG ratio from ELISA was used to classify dengue positive patients into primary and secondary dengue, whereby primary was defined as IgG negative with positive on either IgM, NS1 or RT-PCR; while secondary – positive IgG with negative IgM and positive on either RT-PCR or NS1. If both IgM and IgG were present, IgM/IgG ratio > =1.2 was considered as primary dengue, while < 1.2 - as secondary dengue [6, 16, 21, 22].

### Data analysis

Descriptive analysis was used to describe the sociodemographic and clinical characteristics of the participants. The interrater agreement between the first and second interpreters for each assay in SD Bioline Dengue Duo was assessed using Kappa statistics (k). It was also computed for the agreement of test results between capillary and venous samples for this combo, while ViroTrack Dengue Acute also had additional results for capillary-serum and venous-serum. Agreement was interpreted as poor if k was < 0, slight if 0–0.2, fair if 0.2–0.4, moderate if 0.4–0.6, good if 0.6–0.8, and excellent if 0.8–1.0 [23].

The true positive (TP), false negative (FN), false positive (FP), and true negative (TN) of each index test and various combinations of the components of combo test as compared to the reference standard were used to calculate various diagnostic accuracy parameters and their 95% confidence intervals (95%CI) using standard formulas [24, 25]:

Sensitivity (SN) = TP/(TP + FN);

Specificity (SP) = TN / (TN + FP);

positive predictive value (PPV) = TP / (TP + FP);

negative predictive value (NPV) = TN / (TN + FN);

positive likelihood ratio (LR+) = SN / (1-SP);

negative likelihood ratio (LR-) = (1-SN) / SP;

area under curve (AUC) = (SN + SP) / 2; and.

diagnostic odds ratio (DOR) = (TP/FN) / (FP/TN).

Subgroup analyses by exposure (serotype, day of illness or DOI, dengue infection status) and outcome (lab-confirmed vs presumptive dengue) were also performed to compare sensitivity estimates. For diagnostic utility, post-test probabilities (95%CI) of dengue for positive and negative test were calculated for each test assay and their combinations. For that, pre-test probability (prevalence) of dengue among participants of this study was first converted to odds: prevalence/(1-prevalence). This pre-test odds were then multiplied with corresponding LR to obtain post-test odds that in turn were converted back to probabilities: odds/(1 + odds) [24].

Data analysis was performed using STATA version 12 (StataCorp, TX, US). All inconclusive and missing test results, whether of reference standard or index tests, were excluded from the analysis.

## Results

### Description of the study cohort

Out of the 504 potentially eligible patients who attended the clinic over the study period, 494 (98.1%) agreed to participate in the study. Their age ranged from 11 months to 70.7 years with a mean of 27.2 years (s.d. 11.8). 285 (57.7%) were male. The mean day of fever upon recruitment was 4.3 (s.d. 2.0) days, with a range of 1 to 11 days.

All 494 recruited patients had either capillary and/or venous sample tested on both point-of-care RDT. But only 490 were characterised with the laboratory-based index test - the SD Dengue NS1 Ag ELISA, and all reference tests. Absence of results for any assay was due to insufficient test specimens. The flow of participants for the index tests and their results was presented using STARD diagrams (Figure S1, S2 and S3).

Out of the 490 patients tested with reference tests, 227 were dengue positive, 262 were negative, and 1 was inconclusive. Among the dengue positive patients, 72 were presumptive and 155 were laboratory-confirmed. The latter comprised of 64 positives on both RT-PCR and Panbio NS1 ELISA, 27 positives only on RT-PCR, and 64 positives only on NS1 ELISA.

Among the dengue positive patients, 9 had dengue without warning sign, while 218 reported at least one warning sign according to WHO 2009 classification. One hundred thirty seven of them had primary while 90 had secondary dengue. Lastly, there were 31 DENV-1, 29 DENV-2, 30 DENV-3, and only 1 DENV-4 among the 91 patients tested positive on RT-PCR.

### Agreement between test interpreters and different blood specimens

For SD Bioline NS1 assay, 482 patients had both capillary and venous samples tested and 12 patients had only venous results; while 490 had both results and 4 had only venous result for IgM/IgG assay. For ViroTrack Dengue Acute, 442 patients had both results, while 41 and 11 had either capillary or venous result, respectively. All 494 patients also had serum results for ViroTrack Dengue Acute. Comparison can only be made between test results from different subgroups belonging to the same patient. The kappa and their 95%CI for all comparisons were more than 0.8, indicating excellent agreement (Table S1, S2, S3).

For SD Bioline Dengue Duo, both interpreters almost completely agreed on NS1 assay tested on both capillary and venous samples with kappa of 0.99 (95%CI 0.90–1.00) and 1.00 (95%CI 0.91–1.00), respectively; while the results were not significantly lower for serology tests with point estimates of k ranging from 0.97–0.98 (Table S1). When the final approved test results between capillary and venous were compared, the kappa ranged from 0.96 (95%CI 0.87–1.00) for IgG assay to 1.00 (95%CI 0.91–1.00) for NS1 (Table S2).

For ViroTrack Dengue Acute NS1 assay, the kappa stood at 0.92 (95%CI 0.84–1.00) for capillary-venous, 0.91 (95%CI 0.82–0.99) for capillary-serum, and 0.91 (95%CI 0.83–0.99) for venous-serum (Table S3). These estimates were lower than that of SD Bioline NS1 assay due to the additional EQ category, albeit not statistically significant.

### Diagnostic accuracy of index tests as compared to reference standard

All the diagnostic accuracy parameters were presented in Table 1. Among NS1-only assays, the sensitivity of NS1 ELISA from SD was significantly higher than its own RDT, at 66.5% (95%CI 60.0–72.6) versus 52.4% (95%CI 45.7–59.1), respectively. The sensitivity of ViroTrack Dengue Acute ranked second at 62.3% (95%CI 55.6–68.7) and did not significantly differ from both the above. The specificities were rather comparable, with SD NS1 RDT performed better insignificantly at 97.7% (95%CI 95.1–99.2). There was no significant difference between all three NS1 tests for the other parameters (Table 1).

**Table 1:** Diagnostic accuracy estimates and their 95%CI for all index tests

Parameter	ViroTrack Dengue Acute NS1	SD Dengue NS1 Ag ELISA	SD Bioline DD NS1 only	SD Bioline DD IgM only	SD Bioline DD IgG only	SD Bioline DD NS1 or IgM	SD Bioline DD NS1 or IgM or IgG
Sensitivity^a^, % (95%CI)	*139/223*	*151/227*	*119/227*	*103/227*	*63/227*	*172/227*	*187/227*
	62.3%	66.5%	52.4%	45.4%	27.8%	75.8%	82.4%
	(55.6–68.7)	(60.0–72.6)	(45.7–59.1)	(38.8–52.1)	(22.0–34.1)	(69.7–81.2)	(76.8–87.1)
Specificity^a^, % (95%CI)	*249/262*	*250/262*	*256/262*	*258/262*	*233/262*	*253/262*	*229/262*
	95.0%	95.4%	97.7%	98.5%	88.9%	96.6%	87.4%
	(91.7–97.3)	(92.1–97.6)	(95.1–99.2)	(96.1–99.6)	(84.5–92.5)	(93.6–98.4)	(82.8–91.2)
PPV^a^, % (95%CI)	*139/152*	*151/163*	*119/125*	*103/107*	*63/92*	*172/181*	*187/220*
	91.4%	92.6%	95.2%	96.3%	68.5%	95.0%	85.0%
	(85.8–95.4)	(87.5–96.1)	(89.8–98.2)	(90.7–99.0)	(58.0–77.8)	(90.8–97.7)	(79.6–89.4)
NPV^a^, % (95%CI)	*249/333*	*250/326*	*256/364*	*258/382*	*233/397*	*253/308*	*229/269*
	74.8%	76.7%	70.3%	67.5%	58.7%	82.1%	85.1%
	(69.8–79.4)	(71.7–81.2)	(65.3–75.0)	(62.6–72.2)	(53.7–63.6)	(77.4–86.3)	(80.3–89.2)
LR + (95%CI)	12.6	14.5	22.9	29.7	2.5	22.1	6.5
	(7.32–21.5)	(8.29–25.4)	(10.3–51.0)	(11.1–79.4)	(1.7–3.8)	(11.6–42.1)	(4.7–9.1)
LR - (95%CI)	0.40	0.35	0.49	0.56	0.81	0.25	0.20
	(0.33–0.47)	(0.29–0.42)	(0.42–0.56)	(0.49–0.63)	(0.74–0.89)	(0.20–0.32)	(0.15–0.27)
AUC (95%CI)	0.79	0.81	0.75	0.72	0.58	0.86	0.85
	(0.75–0.82)	(0.78–0.84)	(0.72–0.78)	(0.69–0.75)	(0.55–0.62)	(0.83–0.89)	(0.82–0.88)
DOR (95%CI)	31.7	41.4	47 .0	53.6	3.1	87.9	32.4
	(17.2–58.5)	(22.0–77.9)	(20.5–108.0)	(20.0–143.0)	(1.9–5.0)	(42.8–180.0)	(19.7–53.4)

^a^The italic numbers shown before the parameter estimates are number of correct tests over number of all tests for each corresponding parameter

The sensitivities of both SD serology RDT were lower when compared to all the NS1-only assays, and the difference was significant when compared to ELISA and ViroTrack, with IgM at 45.4% (95%CI 38.8–52.1) and IgG - 27.8% (95%CI 22.0–34.1). However, among all individual assays, IgM had the highest specificity of 98.5% (95%CI 96.1–99.6); while IgG had it lowest - 88.9% (95%CI 84.5–92.5), which was significantly lower when compared to both SD NS1 and IgM RDT. IgG also performed significantly worse in all the other parameters compared to all individual assays except for NPV when compared to IgM. On the contrary, IgM had higher PPV, LR+, and DOR; but worse NPV, LR-, and AUC, when compared to NS1-only assays. Although significant difference was only found in LR- and AUC with ELISA, and only LR- with Viro-Track (Table 1).

When the results of all three individual assays on SD Bioline Dengue Duo were combined, where positive on either one was considered as dengue positive, the diagnostic accuracy parameters generally improved. For SD NS1/IgM combination, all the estimates were better compared to ELISA and ViroTrack, but all these differences were insignificant save for ViroTrack sensitivity, LR-, and AUC. When compared to SD NS1 RDT alone, NS1/IgM performed better except on specificity, PPV, and LR+, with significant difference only in sensitivity, NPV, LR-, and AUC. On the contrary, for SD NS1/IgM/IgG, the insignificant marginal improvement over SD NS1/IgM in sensitivity to 82.4% (95%CI 76.8–87.1), NPV to 85.1% (95%CI 80.3–89.2), and LR- to 0.20 (95%CI 0.15–0.27), was compensated with decrease in all other parameters, which were significant for specificity, PPV, and LR+ (Table 1).

### Sensitivities of index tests in subgroup analyses

The sensitivities of the evaluated index tests stratified into different subgroups were presented in Table 2 and Table 3. When stratified by serotype, all NS1 assays whether alone or in combination had higher sensitivity in detecting DENV-3, followed by DENV-2 and DENV-1. Only the difference between DENV-3 and DENV-1 was significant for ViroTrack, and SD NS1 RDT whether individually or in the form of combo. There was no significant difference between the lower sensitivities of SD serology RDT in the detection of various serotypes, although IgM appeared to do insignificantly better with DENV-1 followed by DENV-3. None of the assays was able to detect the sole DENV-4 patient. When compared between index tests, the sensitivities of all assays with NS1 component did not differ much with each other but were all significantly better than serology-only assays, except between SD NS1 and IgM RDT for DENV-1 (Table 2).

**Table 2:** Sensitivities and their 95%CI for all index tests in different subgroups

Parameter	ViroTrack Dengue Acute NS1	SD Dengue NS1 Ag ELISA	SD Bioline DD NS1 only	SD Bioline DD IgM only	SD Bioline DD IgG only	SD Bioline DD NS1 or IgM	SD Bioline DD NS1 or IgM or IgG
By Serotype							
DENV-1	*20/31*	*20/31*	*14/31*	*7/31*	*3/31*	*19/31*	*20/31*
	64.5 (45.4–80.8)	64.5 (45.4–80.8)	54.8 (36.0–72.7)	22.6 (9.6–41.1)	9.7 (2.0–25.8)	61.3 (42.2–78.2)	64.5 (45.4–80.8)
DENV-2	*23/29*	*23/29*	*24/29*	*3/29*	*3/29*	*25/29*	*27/29*
	79.3 (60.3–92.0)	79.3 (60.3–92.0)	82.8 (64.2–94.2)	10.3 (2.2–27.4)	10.3 (2.2–27.4)	86.2 (68.3–96.1)	93.1 (77.2–99.2)
DENV-3	*29/30*	*28/30*	*28/30*	*5/30*	*3/30*	*29/30*	*29/30*
	96.7 (82.8–99.9)	93.3 (77.9–99.2)	93.3 (77.9–99.2)	16.7 (5.6–34.7)	10.0 (2.1–26.5)	96.7 (82.8–99.9)	96.7 (82.8–99.9)
DENV-4	*0/1*	*0/1*	*0/1*	*0/1*	*0/1*	*0/1*	*0/1*
	0 (0–97.5)	0 (0–97.5)	0 (0–97.5)	0 (0–97.5)	0 (0–97.5)	0 (0–97.5)	0 (0–97.5)
By day of illness							
First 5 days	*102/157*	*109/160*	*93/160*	*51/160*	*33/160*	*117/160*	*128/160*
	65.0 (57.0–72.4)	68.1 (60.3–75.3)	58.1 (50.1–65.9)	31.9 (24.7–39.7)	20.6 (14.6–27.7)	73.1 (65.6–79.8)	80.0 (73.0–85.9)
> = 6 days	*37/66*	*42/67*	*26/67*	*52/67*	*30/67*	*55/67*	*59/67*
	56.1 (43.3–68.3)	62.7 (50.0–74.2)	38.8 (27.1–51.5)	77.6 (65.8–86.9)	44.8 (32.6–57.4)	82.1 (70.8–90.4)	88.1 (77.8–94.7)
By dengue infection status							
Primary	*99/134*	*104/137*	*88/137*	*61/137*	*7/137*	*113/137*	*113/137*
	73.9 (65.6–81.1)	75.9 (67.9–82.8)	64.2 (55.6–72.2)	44.5 (36.0–53.3)	5.1 (2.1–10.2)	82.5 (75.1–88.4)	82.5 (75.1–88.4)
Secondary	*40/89*	*47/90*	*31/90*	*42/90*	*56/90*	*59/90*	*74/90*
	44.9 (34.4–55.9)	52.2 (41.4–62.9)	34.4 (24.7–45.2)	46.7 (36.1–57.5)	62.2 (51.4–72.2)	65.6 (54.8–75.3)	82.2 (72.7–89.5)

The italic numbers shown before the sensitivity estimates are true positives over all disease-positives for the respective assay

**Table 3:** Sensitivities and their 95%CI for all index tests in diagnosing lab-confirmed versus presumptive dengue

Index Tests	Lab-confirmed dengue	Presumptive dengue
ViroTrack Dengue Acute NS1	*117/152*	*22/71*
	77.0 (69.5–83.4)	31.0 (20.5–43.1)
SD Dengue NS1 Ag ELISA	*127/155*	*24/72*
	81.9 (75.0–87.6)	33.3 (22.7–45.4)
SD Bioline DD NS1 only	*106/155*	*13/72*
	68.4 (60.4–75.6)	18.1 (10.0–28.9)
SD Bioline DD IgM only	*56/155*	*47/72*
	36.1 (28.6–44.2)	65.3 (53.1–76.1)
SD Bioline DD IgG only	*25/155*	*38/72*
	16.1 (10.7–22.9)	52.8 (40.7–64.7)
SD Bioline DD NS1 or IgM	*123/155*	*49/72*
	79.4 (72.1–85.4)	68.1 (56.0–78.6)
SD Bioline DD NS1 or IgM or IgG	*127/155*	*60/72*
	81.9 (75.0–87.6)	83.3 (72.7–91.1)

The italic numbers shown before the sensitivity estimates are true positives over all disease-positives for the respective assay

When the analyses were stratified by DOI that usually coincides with day of fever, all NS1-only assays had insignificantly higher sensitivity in the first 5 days compared to later period. In contrast, all serology-only assays performed significantly better in the opposite manner especially for SD IgM RDT. The same trend observed for the serology-only assays was carried forward into both the SD RDT combo, although the difference became insignificant. When compared between all NS1-only assays for the detection of dengue in the first 5 days, ELISA performed insignificantly better than ViroTrack followed by SD RDT. But when the latter was combined with IgM, the sensitivity improved such that the difference became significant compared to itself alone and ViroTrack for DOI > =6 days. The improvement was more substantial for SD NS1/IgM/IgG, where sensitivity was significantly higher versus SD NS1 RDT alone and ViroTrack at any time, and even against ELISA for DOI > =6 days (Table 2).

All NS1-only assays had significantly better sensitivity in detecting primary dengue versus secondary. SD NS1/IgM RDT demonstrated similar trend but the difference was insignificant. This pattern was reverse in SD IgG RDT, while SD IgM RDT and SD NS1/IgM/IgG RDT did not have any within-assay difference between infection status subgroups. When compared between assays with NS1 component, both SD RDT combo assays had significantly better sensitivity compared to SD NS1 RDT alone for both primary and secondary dengue, while the sensitivity of SD NS1/IgM/IgG RDT was also significantly better than ViroTrack and ELISA in detecting secondary dengue thanks to the additional IgG component. Serology-only assays had significantly lower sensitivity compared to all other assays with NS1 component in detecting primary dengue, while SD IgG RDT was also significantly less sensitive compared to SD IgM RDT. However, for secondary dengue, SD IgG RDT performed better than all individual assays and significantly better than SD NS1 RDT (Table 2).

Similar to the trend observed above, all NS1-only assays and SD NS1/IgM RDT were more sensitive in the detection of laboratory-confirmed over presumptive dengue, albeit without significant difference. On the contrary, both serology-only assays performed significantly better in detecting presumptive over laboratory-confirmed dengue. Again, SD NS1/IgM/IgG RDT performed equally well in both with sensitivity point estimates above 80%. This combination was an all-rounder with significantly higher sensitivity compared to serology-only assays in the detection of lab-confirmed dengue owing to its NS1 component, and significantly higher sensitivity compared to all NS1-only assays in the detection of presumptive dengue owing mainly to its IgM component (Table 3).

### Diagnostic utility of index tests

The pre-test probability of dengue (or proportion of dengue patients among all patients) was 46.3% in this study (Table 4). All index tests if tested positive would increase the probability of dengue diagnosis to above 80%, except SD IgG that registered only 68.2% (95%CI 58.8–76.1), which was significantly lower than all other assays. The highest post-test probability of dengue for positive test was achieved by SD IgM RDT at 96.2% (95%CI 90.4–98.5), followed by SD NS1 RDT and SD NS1/IgM RDT at 95.1% (95%CI 89.8–97.7) and 94.9% (95%CI 90.8–97.3), respectively. These figures achieved by SD RDT NS1 or/and IgM components were significantly higher than the 84.8% (95%CI 80.1–88.5) attained by its NS1/IgM/IgG combination. The results for SD ELISA and ViroTrack did not differ significantly between each other nor with other assays, except with SD IgG RDT (Table 4).

**Table 4:** Diagnostic utility estimates and their 95%CI for all index tests

Parameter	ViroTrack Dengue Acute NS1	SD Dengue NS1 Ag ELISA	SD Bioline DD NS1 only	SD Bioline DD IgM only	SD Bioline DD IgG only	SD Bioline DD NS1 or IgM	SD Bioline DD NS1 or IgM or IgG
Post-test probability of dengue for positive test, % (95%CI)	91.5	92.5	95.1	96.2	68.2	94.9	84.8
	(86.2 - 94.8)	(87.6 - 95.6)	(89.8 - 97.7)	(90.4 - 98.5)	(58.8 - 76.1)	(90.8 - 97.3)	(80.1 - 88.5)
Post-test probability of dengue for negative test, % (95%CI)	25.3	23.0	29.3	32.1	40.9	17.6	14.7
	(22.2 - 28.6)	(19.9 - 26.5)	(26.5 - 32.2)	(29.5 - 34.8)	(38.7 - 43.1)	(14.5 - 21.2)	(11.4 - 18.6)

Pre-test probability = 46.3%

When it comes to post-test probability of dengue for negative test, SD NS1/IgM/IgG RDT performed best with 14.7% (95%CI 11.4–18.6), significantly lower than all individual assays. SD NS1/IgM RDT came in second at 17.6% (95%CI 14.5–21.2), also significantly better than all individual assays except SD ELISA. The latter had the best result among all individual assays followed by ViroTrack and SD NS1 RDT, at 23.0% (95%CI 19.9–26.5), 25.3% (95%CI 22.2–28.6), and 29.3% (95%CI 26.5–32.2), respectively. Serology-only assays performed significantly worse than all NS1-only assays in post-test probability of negative test, with SD IgG RDT also performed significantly worse than SD IgM RDT at 32.1% (95%CI 29.5–34.8) versus 40.9% (95%CI 38.7–43.1), respectively (Table 4).

## Discussion

Most published dengue RDT evaluation studies used serum samples. Some studies also used whole blood specimen and only one used capillary blood [26–29]. The excellent agreement between the results tested on capillary, venous, and serum samples in this study demonstrated that all three of them can be used on RDT, provided that anticoagulant-coated tool is used for the collection of whole blood specimen. The validity of results from capillary blood has practical implication when minimal invasiveness and/or rapidity is required such as in young children or during massive dengue outbreak.

No study was published prior to this for the evaluation of ViroTrack Dengue Acute, while SD Dengue NS1 Ag ELISA and SD Bioline Dengue Duo have been extensively evaluated. The individual components of the SD Bioline RDT had point estimates of sensitivity and specificity for the diagnosis of acute dengue that ranged within 44.4–94.9% and 70.9–100.0% for NS1 [30–32], 10.0–98.0% and 66.0–100.0% for IgM [18, 32, 33], and 38.8–90.1% and 92.5–100.0% for IgG [22, 34, 35], respectively. On the other hand, the point estimates of sensitivity and specificity of SD Dengue NS1 Ag ELISA published previously were 55.2–76.8% and 94.6–98.6%, respectively [15, 23, 36]. The results in this study fell within the above range except for the IgG component of the SD RDT, where both sensitivity and specificity were lower than previously found. This difference may be attributed to the underlying difference in study design, patient population, definition of reference standard, and other study characteristics [37–39].

Since differences in study characteristics would modify the outcomes, it is difficult to directly compare diagnostic accuracy and utility between different studies without proper assessment. In other words, the apparent difference in diagnostic accuracy and utility parameters between two tests evaluated in two different studies may be due to the difference between their study characteristics instead of the actual performance of the tests themselves. However, diagnostic tests evaluated within the same study on the same patient population under the same condition can be directly compared [37]. In this study, among all the individual assays, SD Dengue NS1 Ag ELISA had the best performance followed by ViroTrack, SD Bioline Dengue Duo NS1, IgM and IgG components. This outcome is expected as both ELISA and ViroTrack employed objective result read-out based on physical properties of light transmission in contrast to the subjective interpretation of SD RDT [8, 13–15, 18, 30].

The sensitivities of all the evaluated assays turned out as expected in the subgroup analyses. The NS1-only assays performed better in detecting dengue infection in the first 5 days versus 6 days and above as NS1 antigen is actively produced and secreted in the first week [7, 18, 22, 29, 30, 40–43]. However, the decline in performance was more obvious for SD NS1 RDT as reducing level of NS1 towards the end of the week might produce a test line too faint to be detected with naked eyes [8]. On the contrary, serology-only assays performed better after day 5 when IgM and IgG started to be secreted to counter the infectious agent [5, 7, 17, 18, 22, 29, 30, 40–44]. The observation that SD IgM performed better than IgG, especially after day 5, is most likely due to the use of IgM ELISA in the definition of the reference standard.

This study discovered that NS1-only assays had higher sensitivity in detecting primary and laboratory-confirmed dengue, while serology-only assays were better in secondary and presumptive dengue. Again, the reason for this lies predominantly in the definition of reference standard, where primary and laboratory-confirmed dengue were mostly those tested positive on RT-PCR and/or Panbio NS1 ELISA; while secondary and presumptive were more dependent on IgM and IgG capture ELISA for their definitions. This similar trend was observed repeatedly in previous studies with some variations attributable to differences in study characteristics [18, 23, 29, 31–33, 35, 36, 40].

The finding that all NS1-only index tests had lower sensitivity while SD IgM RDT had it higher for the detection of DENV-1 as compared to other serotypes was unexpected. Some previous studies did not demonstrate difference [27, 45], while others did [18, 30, 31, 46, 47]. Further analysis (data not shown) showed that there were more patients beyond the 5th day of illness among those with DENV-1, which may partially explain these results. On the other hand, the failure of all assays to detect the sole DENV-4 was probably due to chance, as this patient had secondary dengue (with low IgM level) on 5th day of illness, when the level of free and detectable IgG and NS1 happened to be too low after their union in vivo [23, 45, 47, 48]. Lastly, the serotype distribution found in this study agreed with previous finding [49].

Notwithstanding the underperformance of SD Bioline Dengue Duo individual components, the diagnostic accuracy drastically improved when they were combined and interpreted as dengue positive if found positive on either one component. Combination of SD NS1 and IgM improved sensitivity, NPV and LR- (better rule-out test if found negative) without compromising on the other parameters, while the addition of IgG component further improved the rule-out parameters at the expense of rule-in parameters. This trend was in line with previous findings [15, 23, 27, 29, 30, 32, 34, 35, 40, 41, 50, 51]. The repercussion of this finding is that dengue combo test is always superior to individual assay RDT as combo has the ability to detect dengue infection regardless of the phase of illness [40, 41].

This recommendation is further backed up by the diagnostic utility from this study. As shown in Table 4, tested positive for SD NS1/IgM RDT was able to double the probability of dengue infection in a patient to be more than 90%, leaving only less than 10 out of 100 wrongly diagnosed non-dengue patients. Although all individual assays except SD IgG RDT were able to more or less match this performance, they could not reproduce the same when it came to negative tests. If a patient was tested negative for any of the individual assays, the best post-test probability of dengue was still 23.0%. In other word, at least 23 out of 100 dengue patients were FN that might have been misdiagnosed. In contrast, SD combo tests were able to reduce FN to just 15 to 18 per 100 dengue patients. While dengue FP may not be a big concern due to its small proportion and relatively less harmful supportive treatment unless in the case of misdiagnosis of other more severe diseases, high number of FN might lead to late diagnosis and delayed administration of required life-saving treatment for dengue patients. As such, in a clinical setting, especially in primary care, it is important for a dengue RDT to act as a screening tool that can detect more cases with minimal FN.

Although this study found that SD Bioline Dengue Duo, a dengue RDT based on immunochromatographic principle, was a more preferred tool when used as a combo test; the new biosensors-based ViroTrack Dengue Acute was not without its own advantages. It requires only 30 mcl of blood specimen compared to 100 mcl required for SD NS1 RDT. This feature is important when blood sampling is difficult and yield is scarce, such as among paediatric patients. Its test procedure requires minimal training, and is as simple and user-friendly as that of SD RDT at least in terms of sample loading, but its test duration is slightly shorter. Still, after loading blood sample, ViroTrack must be inserted into a Blubox, an electronic device that runs on electricity. This may create a bottleneck if more than one sample must be run concurrently, unless commercialised version of Blubox comes with the ability to run multiple tests. Yet, the presence of Blubox increases test sensitivity and reduces number of false positive from delayed interpretation common for immunochromatographic tests [8]. Furthermore, its ability to connect with local and global network reduces the rate of manual transcription error in reporting [52], and makes results available immediately upon diagnosis to treating doctors and public health authority for quick intervention [53]. Nevertheless, ViroTrack must first be available in the form of a combo test and proven to be more accurate, cost-effective, and beneficial in future studies for this new technology to be popularised.

The strength of this study lies in its sound methodology and application. As mentioned above, it is difficult to directly compare diagnostic performance between evaluation studies due to different study characteristics [37]. In the same way, it is also fundamentally incorrect to directly apply their results into daily practice or for policy making. Good performances reported in case-control or laboratory-based studies may be due to biases instead of the discriminatory power of the evaluated tests [7, 39]. In contrast, the cross-sectional prospective design in an actual primary care clinical setting seen in this study produced a more realistic set of diagnostic performance parameters that is true to other similar clinical settings, making the application of its results easier and more valid. Besides, it complied with STARD-guidelines for quality assurance [10]. In addition, diagnostic utility presented here is more intelligible to clinical practitioners and policy makers compared to the usually reported diagnostic accuracy. With simple calculation based on the formulas provided here or using rough estimation, the diagnostic utility of the three index tests evaluated in this study can be estimated for any clinical setting [24, 54]. However, it should be cautioned that this exercise took into account only dengue diagnostics without consideration of other diseases. Practitioners should also use existing clinical reasoning for differential diagnosis.

In Malaysia, four previously conducted dengue RDT evaluation studies were exclusively laboratory-based and only one employed cross-sectional prospective design [18, 41, 55, 56]. This study was the first conducted prospectively among consecutively sampled patients in primary care setting. It provided better and more updated insight on the application of dengue RDT in Malaysia. Furthermore, it was the first that evaluated a biosensors-based RDT in this setting and compared it with extensively used RDT and NS1 ELISA for a more comprehensive understanding of their relative performance, which is absent in most other studies that evaluated only either RDT or ELISA. Two limitations of this study are, first, only single sample was collected from each patient, making the reference standard based on serology presumptive rather than conclusive [6, 20, 39]; and second, the diagnostic utility calculated was based on the pre-test probability of disease based on the WHO 2009 guidelines without taking into account of the haematological result. Nevertheless, these limitations perfectly reflect the actual situation faced by clinicians in the front line, making the study results more realistic and applicable to the real-world circumstances.

## Conclusions

In conclusion, for the diagnosis of acute dengue infection, new biosensors-based dengue RDT such as ViroTrack Dengue Acute performed almost as well as SD Dengue NS1 Ag ELISA, while the latter was superior to SD Bioline NS1 assay. The addition of serology component to SD Bioline Dengue Duo, however, greatly enhanced its diagnostic accuracy and utility parameters almost beyond that of SD Dengue NS1 ELISA, making it a better point-of-care dengue diagnostic tool as it would miss the fewest dengue patients. As such, ViroTrack Dengue Acute can be a potential alternative to existing combo RDTs only if its combination with serology components matches or outperforms them in diagnosing dengue. This decision making should be based on the results of properly conducted diagnostic test accuracy and economic evaluation studies in the future that allow within-study comparison of multiple diagnostic tools instead of comparing between heterogeneous studies.

## Supplementary information

**Additional file 1: Figure S1.** STARD flow diagram for ViroTrack Dengue Acute.

**Additional file 2: Figure S2.** STARD flow diagram for SD Dengue NS1 Ag ELISA.

**Additional file 3: Figure S3.** STARD flow diagram for SD Bioline Dengue Duo.

**Additional file 4: Table S1.** Interrater agreements and their 95% CI between two interpreters for capillary and venous specimens tested on different assays of SD Bioline Dengue Duo. **Table S2.** Agreements and their 95% CI between the results of capillary and venous specimens tested on different assays of SD Bioline Dengue Duo. **Table S3.** Agreements and their 95% CI between the results of different specimens tested on ViroTrack Dengue Acute.

## Data Availability

The datasets used and/or analysed during the current study, as well as the study protocol, are available from the corresponding author on reasonable request.

## Abbreviations

95%CI: 95% confidence intervals
AUC: Area under curve
DD: Dengue duo
DENV: Dengue virus
DOI: Day of illness
DOR: Diagnostic odds ratio
ELISA: Enzyme-linked immunosorbent assay
EQ: Equivocal
FN: False negative
FP: False positive
HI: Haemagglutination inhibition
IgG: Immunoglobulin G
IgM: Immunoglobulin M
k: Kappa statistics
LR +: Positive likelihood ratio
LR-: Negative likelihood ratio
MNPs: Magnetic nanoparticles
NPV: Negative predictive value
NS1: Non-structural antigen-1
PPV: Positive predictive value
RDT: Rapid diagnostic test
RT-PCR: Reverse transcription-polymerase chain reaction
s.d.: Standard deviation
SD: Standard Diagnostics
SN: Sensitivity
SP: Specificity
STARD: Standards for the reporting of diagnostic accuracy studies
TN: True negative
TP: True positive
VI: Virus isolation
WHO: World Health Organisation
