# Supplementary figures and images for: Diagnostic accuracy and utility of three dengue diagnostic tests for the diagnosis of acute dengue infection in Malaysia
Source: BMC Infect Dis. 2020 Mar 12;20:210. doi: 10.1186/s12879-020-4911-5 (PMC7069157; doi:10.1186/s12879-020-4911-5)

Figure S1 STARD flow diagram for ViroTrack Dengue Acute


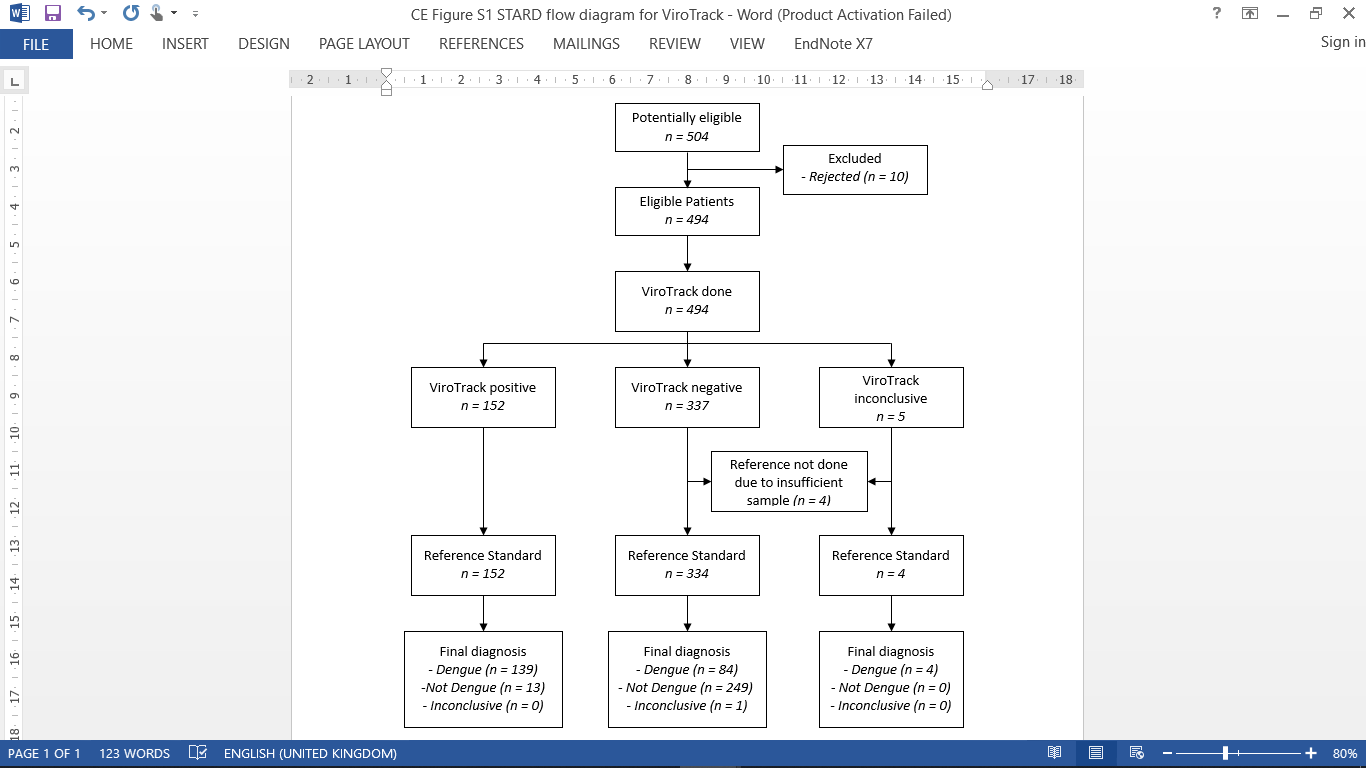

Supplement: Supplementary file 1 — Additional file 1: Figure S1. STARD flow diagram for ViroTrack Dengue Acute. [file 12879_2020_4911_MOESM1_ESM.docx]

Figure S2 STARD flow diagram for SD Dengue NS1 Ag ELISA


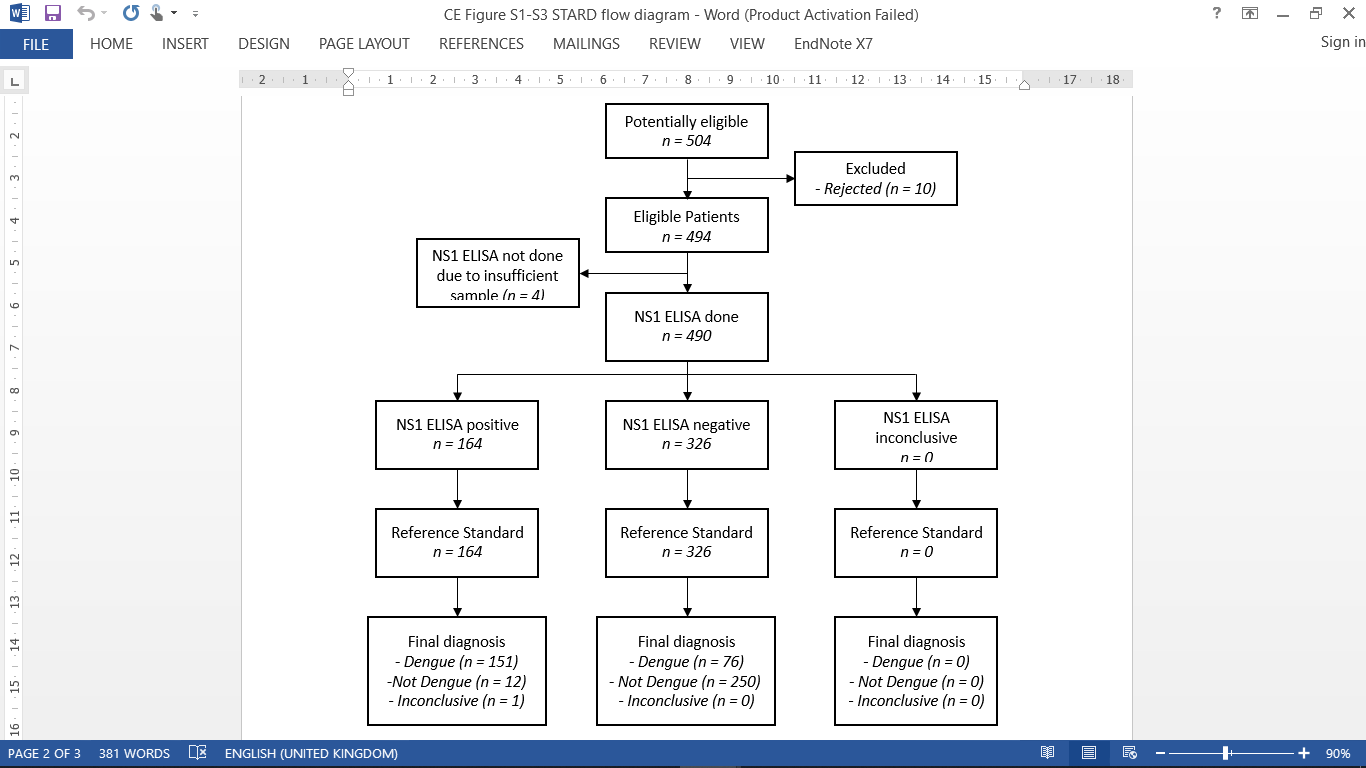

Supplement: Supplementary file 2 — Additional file 2: Figure S2. STARD flow diagram for SD Dengue NS1 Ag ELISA. [file 12879_2020_4911_MOESM2_ESM.docx]

Figure S3 STARD flow diagram for SD Bioline Dengue Duo


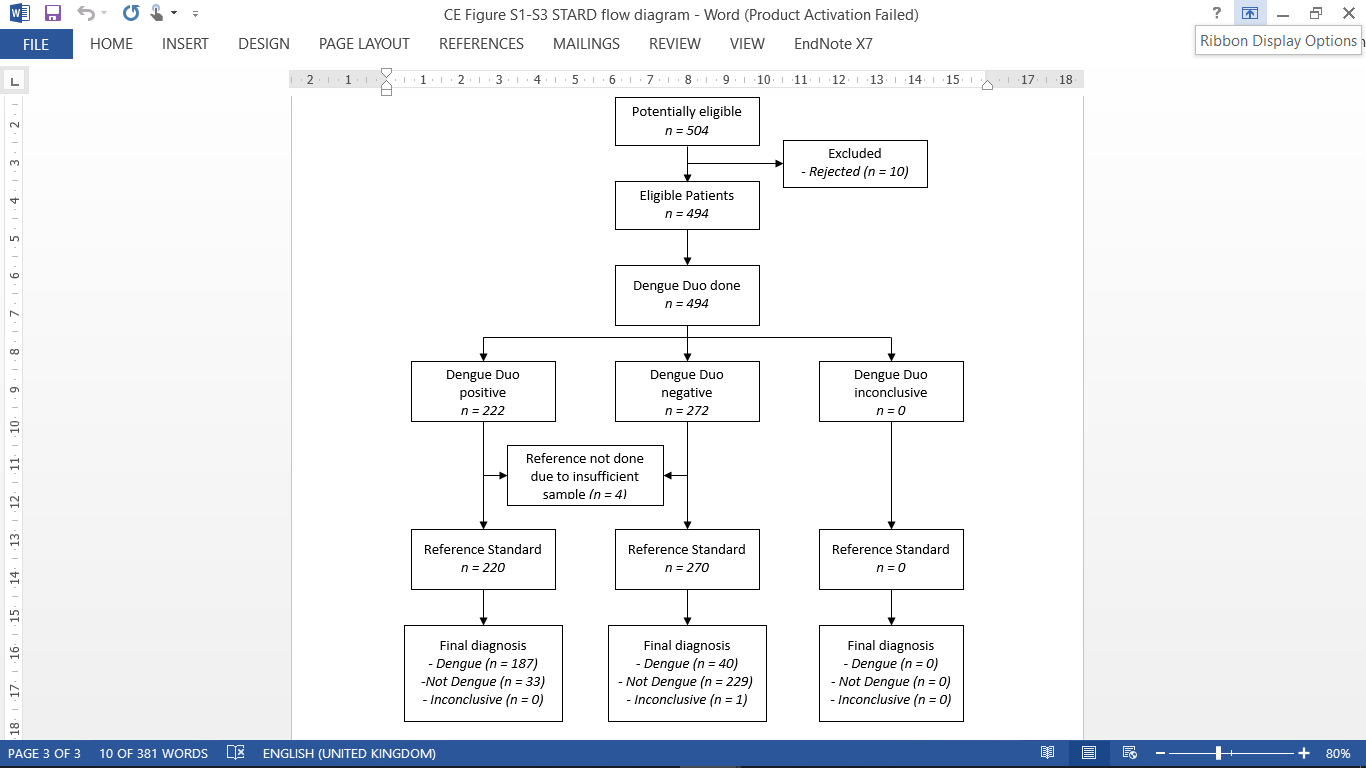

Supplement: Supplementary file 3 — Additional file 3: Figure S3. STARD flow diagram for SD Bioline Dengue Duo. [file 12879_2020_4911_MOESM3_ESM.docx]
