# Supplementary material for: Diagnostic accuracy and utility of three dengue diagnostic tests for the diagnosis of acute dengue infection in Malaysia
Source: BMC Infect Dis. 2020 Mar 12;20:210. doi: 10.1186/s12879-020-4911-5 (PMC7069157; doi:10.1186/s12879-020-4911-5)
Supplement: Supplementary file 4 — Additional file 4: Table S1. Interrater agreements and their 95% CI between two interpreters for capillary and venous specimens tested on different assays of SD Bioline Dengue Duo. Table S2. Agreements and their 95% CI between the results of capillary and venous specimens tested on different assays of SD Bioline Dengue Duo. Table S3. Agreements and their 95% CI between the results of different specimens tested on ViroTrack Dengue Acute. [file 12879_2020_4911_MOESM4_ESM.docx]

Table S1 Interrater agreements and their 95% CI between two interpreters for capillary and venous specimens tested on different assays of SD Bioline Dengue Duo

| Specimen Type | Assay Type | Kappa (95% CI) |
| --- | --- | --- |
| Capillary | NS1 | 0.99 (0.90 - 1.00) |
|  | IgM | 0.98 (0.89 - 1.00) |
|  | IgG | 0.97 (0.88 - 1.00) |
| Venous | NS1 | 1.00 (0.91 - 1.00) |
|  | IgM | 0.97 (0.88 - 1.00) |
|  | IgG | 0.98 (0.89 - 1.00) |

Table S2 Agreements and their 95% CI between the results of capillary and venous specimens tested on different assays of SD Bioline Dengue Duo

| Compare between | Assay Type | Kappa (95% CI) |
| --- | --- | --- |
| Capillary-Venous | NS1 | 1.00 (0.91 - 1.00) |
|  | IgM | 0.98 (0.89 - 1.00) |
|  | IgG | 0.96 (0.87 - 1.00) |

Table S3 Agreements and their 95% CI between the results of different specimens tested on ViroTrack Dengue Acute

| Compare between | Kappa (95% CI) |
| --- | --- |
| Capillary-Venous | 0.92 (0.84 - 1.00) |
| Capillary-Serum | 0.91 (0.82 - 0.99) |
| Venous-Serum | 0.91 (0.83 - 0.99) |
